# A Methodology to Quantify the Geometrical Complexity of the Abdominal Aortic Aneurysm

**DOI:** 10.1038/s41598-019-53820-z

**Published:** 2019-11-22

**Authors:** Faidon Kyriakou, William Dempster, David Nash

**Affiliations:** Department of Mechanical and Aerospace Engineering, University of Strathclyde, 75 Montrose Street, Glasgow, G1 1XJ UK

**Keywords:** Cardiovascular diseases, Medical research, Biomedical engineering

## Abstract

The abdominal aortic aneurysm (AAA) anatomy influences the technical success of the endovascular aneurysm repair (EVAR), yet very few data regarding the aortic tree angles exist in the literature. This poses great limitations in the numerical analyses of endografts, constraining their design improvement as well as the identification of their operational limitations. In this study, a matrix Φ of 10 angles was constructed for the description of the pathological region and was implemented on a large dataset of anatomies. More specifically, computed tomography angiographies from 258 patients were analysed and 10 aortic angles were calculated per case, able to adequately describe the overall AAA shape. 9 dimensional variables (i.e. diameters and lengths) were also recorded. The median and extreme values of these variables were computed providing a detailed quantification of the geometrical landscape of the AAA. Moreover, statistical analysis showed that the identified angles presented no strong correlation with each other while no lateral or anterior/posterior symmetry of the AAA was identified. These findings suggest that endograft designers are free to construct any extreme case-studies with the values provided in a mix-and-match manner. This strategy can have a powerful effect in EVAR stent graft designing, as well as EVAR planning.

## Introduction

Abdominal aortic aneurysm (AAA) is the permanent and irreversible dilatation of the lower section of the aorta. In AAAs, the vessel expands becoming susceptible to rupture, a highly dangerous incident which may lead to death. Prevention or emergent intervention of rupture has been conducted for decades via open surgery but in recent years the approach of endovascular aneurysm repair (EVAR) is preferred^1^.

In clinical practice, several methods for defining, grading and evaluating the aneurysmal site have been proposed^2–6^, but with small variations, all of them take into account the following anatomical characteristics:

1. Proximal aortic neck
  morphology (straight, taper, reverse taper or bulging)
  length
  diameter
  angle between the flow axis of the neck and the body of the aneurysm
  amount of thrombus and calcification
2. Aneurysm
  maximum AAA diameter
  tortuosity
  most acute aortic angle
  amount of thrombus and calcification
3. Distal aortic neck
  length
  diameter
4. Iliac arteries (common, internal, external)
  diameter
  evaluation of the presence of stenosis/occlusion
  sealing zone length
  tortuosity
  most acute aortic angle
  amount of thrombus and calcification.

Apart from the aneurysm and the aortic necks, which are self-evidently necessary to be examined, iliacs are also included in all reports, because when ill-shaped they can be an exclusion criteria for EVAR. While the overall most common factor of EVAR disqualification is regarded to be the length and anatomy of the proximal AAA neck^7,8^, it is calcification, occlusion and tortuosity of the iliac arteries that are responsible for the majority of access complications during EVAR procedures^9^. Despite that, quantification of the angles involved in the AAA is rarely performed in clinical routine^9^. According to Henretta *et al*.^10^, the difficult anatomy of the iliac arteries can lead to some form of complication (injury of the arteries, misalignment of the stent-graft during deployment etc.) in up to 47% of patients whereas according to Clough *et al*.^11^, up to 17% of all EVAR procedures can result in significant problems.

Nowadays, it is common knowledge that AAA anatomy influences EVAR technical success, endoleak rate, migration of the endograft, as well as the need for secondary interventions^4^. It is for these reasons that the identification of average and extreme/worst case values for the AAA geometry is paramount, in order to challenge endograft designs in experimental set-ups, before they even reach the operating theatre. Nevertheless, similar to clinical practice, only a few angles are discussed in the literature, while global metrics like the tortuosity index cannot describe the geometry in a unique way that can allow its recreation.

Numerical analyses that try to establish how the variability in aortic geometry affects the endografts, or whether specific aortic angles result in endovascular complications, have to assume ranges of values for variables ill-documented. Li *et al*. reported the aortic neck angle doesn’t affect the migration force of the stent when smaller than 30°, yet for values above 40°, the force can almost triple^12^. They also demonstrated that the neck angle significantly influences the blood flow of an unstented AAA. Similarly, Morris *et al*.^13^ reported that increase in the aortic bifurcation angle increases the stent graft’s drag force by up to 50%, without establishing how common the angle values they used are. Furthermore, these parametric studies usually occur in a single plane, meaning that complex aortic angle changes in realistic 3D structures are avoided.

In this study, a series of measurements and statistical analyses were made based on a large dataset of EVAR patients. The aim was to quantify an extended set of geometrical variables for the AAA. These results can help stent graft investigators build both representative and challenging case studies for the assessment and improvement of endograft devices. In addition, the methodology presented for the description of the AAA shape can be used as a measurement protocol during EVAR planning, aiding to the identification of the most appropriate endograft given the arterial geometry of a patient and the endografts’ optimum operational range.

## Methods

### Study population and data acquisition

**Figure 1:**
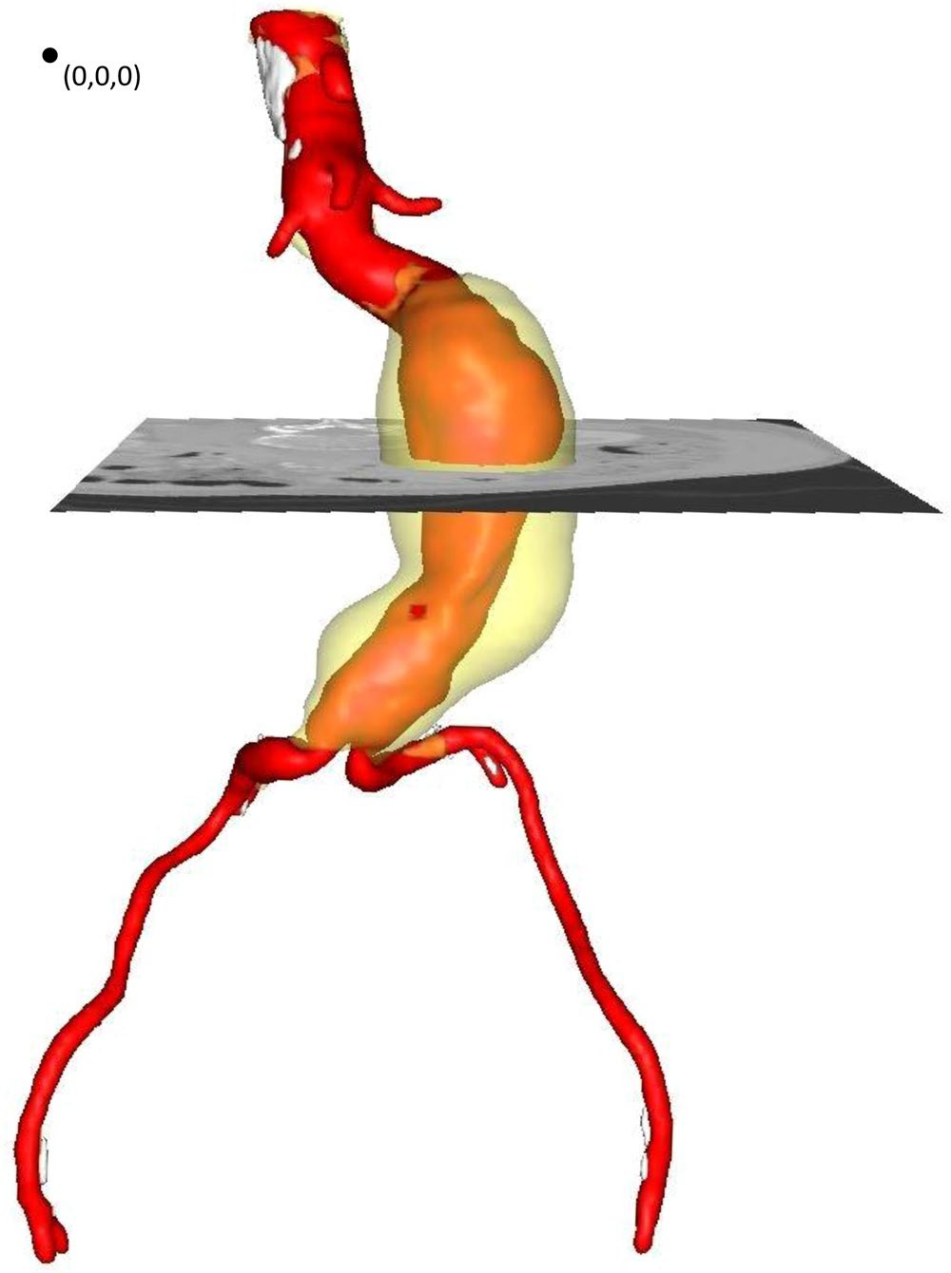
3D reconstruction of the aortic geometry from 2D CTA scans in the m2s Preview. The blood lumen (red), the calcified regions (white) and the thrombus (transparent yellow) are visible. The dot at the top left corner corresponds to the (0,0,0) point of the coordinate system, used for all measurements. It is located at the posterior right corner of the 1st CTA scan.

A series of data provided by M2S, Inc. West Lebanon, NH, USA were used to examine a range of aortic characteristics (Fig. 1). Computed tomography angiography (CTA) scans were originally collected during the “Vascutek Anaconda stent graft system phase II IDE study” in the period May 2009 to July 2011 in various sites in the United States and Canada. The study was approved by the U.S. Food and Drug Administration (registration number: NCT00612924 in the U.S. National Library of Medicine^14^) in accordance with relevant regulations. All patients provided their informed consent. Those data were supplied to us anonymised, with age and gender being the only descriptors of the patients.

The study included 258 patients (222 (86%) men, 34 (13%) women and 2 (1%) unspecified) aged between 51 and 88 years old. It was a non-randomized, continuous enrolment study that included patients treated with the Anaconda^TM^ stent graft (Terumo Aortic). Patients provided their consent for the use of their data and the inclusion criteria for them were:

- Infrarenal AAA ≥ 4.0 cm in diameter, or AAA growth ≥ 1.0 cm/year
- Iliac artery distal fixation sites ≥ 20 mm in length
- Ability to preserve at least one internal iliac artery
- Femoral/Iliac artery’s size and morphology should be compatible with the appropriate delivery system (18 F, 20 F or 23 F).

Pregnant patients, or patients with thrombus, calcification and/or plaque ≥ 2 mm in thickness and/or 50% continuous coverage of the vessel’s circumference in the intended fixation site were excluded. Among the exclusion criteria were also aneurysms that extended above the renal arteries, significant (>80%) renal artery stenosis not readily treatable, previous AAA repairs and ruptured or leaking AAAs.

The follow up period of the study was 5 years, but herein only pre-op data were used.

### Aortic angles

**Figure 2:**
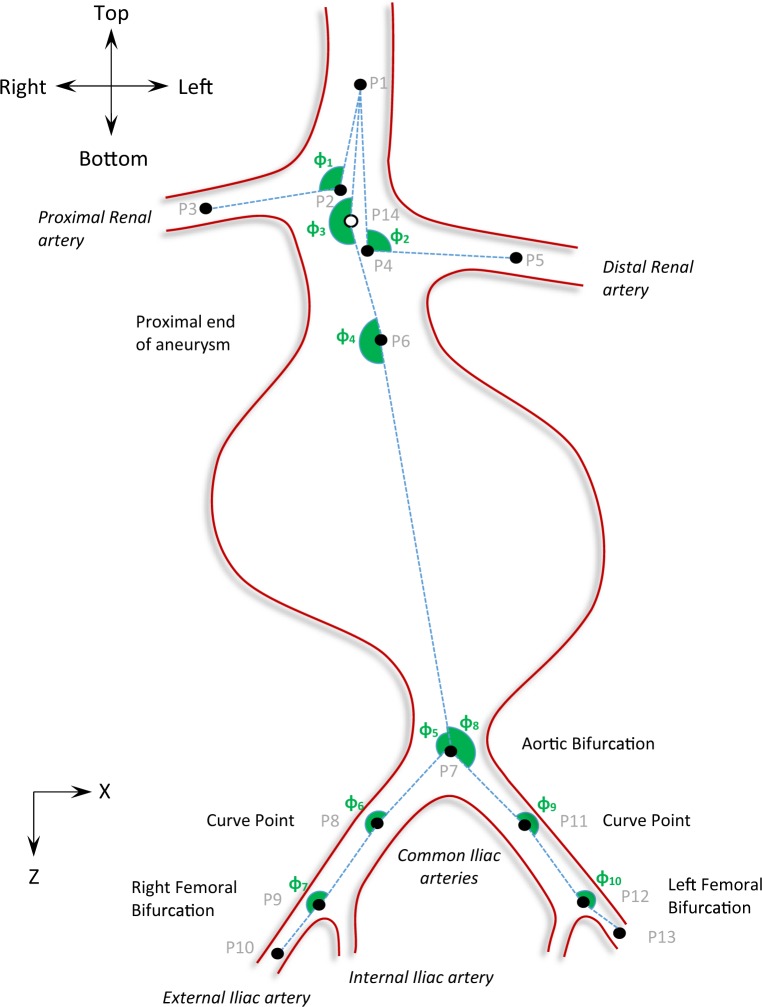
Schematic of the front view of an AAA. XZ-plane. 14 points are identified on the centreline of the aneurysm that allow the definition of 10 angles. Positive angles are illustrated with green.

In the process of describing the AAA shape, a set of 10 angles was used to accurately describe the pathological region, using the m2s Preview v4.0.1 software. More specifically, following the centreline of the vascular tree, 14 points were identified as critical to describe the AAA geometry (Fig. 2 and Table 1). After qualitatively studying a series of AAAs, these points were considered both strategic and adequate to “depict” the overall AAA shape. Note that since tortuosity at the aneurysmal region is very rare^15^, no points in the aneurysmal sac were considered. Furthermore, even if the aneurysm does exhibit such an angle, the endograft is unlikely to follow it, making it, therefore, irrelevant for the current study.

**Table 1:** The 14 critical points used to describe the AAA’s geometry.

P1	At the centre of the aorta, a few centimetres above the renals, usually at the superior mesenteric region.
P2	At the centre of the aorta, at the origin of the proximal renal artery.
P3	At the centre of the proximal renal artery, a few centimetres further from its origin (P2), at a suitable distance away from the junction.
P4	At the centre of the aorta, at the origin of the distal renal artery.
P5	At the centre of the distal renal artery, a few centimetres further from its origin (P4), at a suitable distance away from the junction.
P6	At the centre of the aorta, at the proximal end of the aneurysm.
P7	At the centre of the aortic bifurcation.
P8	At the centre of the right common iliac artery, at the most acute curve point.
P9	At the centre of the right femoral bifurcation.
P10	At the centre of the right external iliac artery, a few centimetres further from the bifurcation.
P11	At the centre of the left common iliac artery, at the most acute curve point.
P12	At the centre of the left femoral bifurcation.
P13	At the centre of the left external iliac artery, a few centimetres further from the bifurcation.
P14	The midpoint between points P2 and P4.

**Figure 3:**
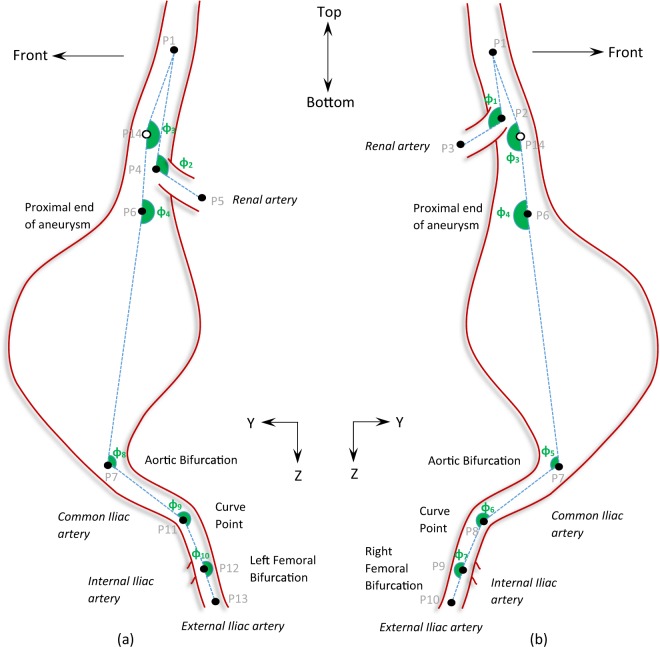
Schematic of the left (**a**) and right (**b**) view of an AAA. YZ-plane. Positive angles are illustrated with green.

**Figure 4:**
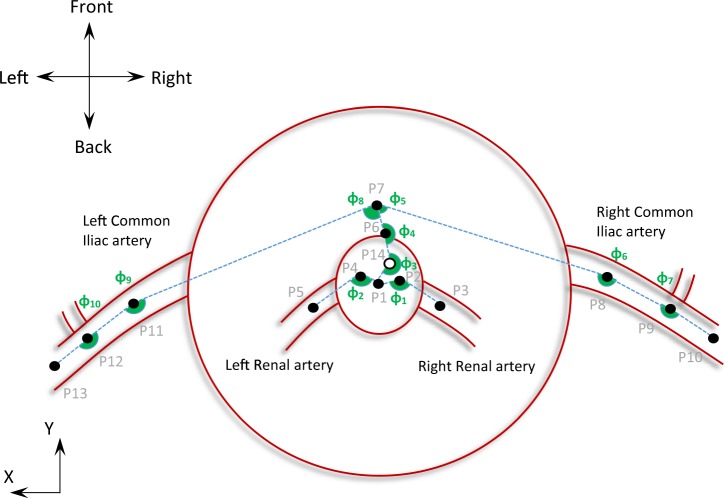
Schematic of the top view of an AAA. XY-plane. Positive angles are illustrated with green.

Points were manually selected on the CTA scans for all patient datasets, and their assigned coordinates were based on the inherent coordinate system of each scan set (Fig. 1). Subsequently, using these 14 points and an in-house Matlab algorithm (version R2015b, MathWorks), 10 angles were calculated in space, as well as their projections on the frontal, the sagittal and the transverse planes (Figs. 2–4 and Table 2), hereafter mentioned as XZ, YZ and XY planes respectively. The advantage of this strategy lies at the ease of identifying the points of interest on the CTA scans. Rather than directly measuring the angles, we limited ourselves into identifying points, reducing the interpretation error. The same process is used in specialized, commercial software as well (e.g. EndoSize®, Therenva SAS, France), yet only for a couple of angles and only in 3D space.

**Table 2:** The 10 angles used to specify the AAA.

*φ*_1_	from the triad of points (P1, P2, P3)
*φ*_2_	from the triad of points (P1, P4, P5)
*φ*_3_	from the triad of points (P1, P14, P6)
*φ*_4_	from the triad of points (P14, P6, P7)
*φ*_5_	from the triad of points (P6, P7, P8)
*φ*_6_	from the triad of points (P7, P8, P9)
*φ*_7_	from the triad of points (P8, P9, P10)
*φ*_8_	from the triad of points (P6, P7, P11)
*φ*_9_	from the triad of points P7, P11, P12)
*φ*_10_	from the triad of points (P11, P12, P13)

The angles for all patients were calculated and the median and range of them documented. Note that in accordance to the literature, when calculating the angles in space, angles are always ≤180°. The same is not true, though, for the projected angles. The projections of the angles on the XZ, YZ and XY planes may be >180°; Figs. 2–4 help define their positive direction.

As a consequence of the analysis, a patient’s aortic shape can be concisely reported as a 1 × 10 matrix ***Φ***:

1 $${{\boldsymbol{\Phi }}}_{3{\boldsymbol{D}}}=({\phi }_{1}\,{\phi }_{2}\,{\phi }_{3}\,{\phi }_{4}\,{\phi }_{5}\,{\phi }_{6}\,{\phi }_{7}\,{\phi }_{8}\,{\phi }_{9}\,{\phi }_{10})$$

using 3D space angle values, for the clinical practice. Similarly, a 3 × 10 matrix that reports the projection of these angles in 3 planes could be used to describe the aortic tree shape for computer-aided design (CAD) modelling in bioengineering applications:

2 $${{\boldsymbol{\Phi }}}_{{\boldsymbol{Proj}}}=(\,\begin{array}{c}{\phi }_{1}^{{{\rm X}}{{\rm Z}}}\,{\phi }_{2}^{{{\rm X}}{{\rm Z}}}\,{\phi }_{3}^{{{\rm X}}{{\rm Z}}}\,{\phi }_{4}^{{{\rm X}}{{\rm Z}}}\,{\phi }_{5}^{{{\rm X}}{{\rm Z}}}\,{\phi }_{6}^{{{\rm X}}{{\rm Z}}}\,{\phi }_{7}^{{{\rm X}}{{\rm Z}}}\,{\phi }_{8}^{{{\rm X}}{{\rm Z}}}\,{\phi }_{9}^{{{\rm X}}{{\rm Z}}}\,{\phi }_{10}^{{{\rm X}}{{\rm Z}}}\\ {\phi }_{1}^{{\Upsilon }{{\rm Z}}}\,{\phi }_{2}^{{\Upsilon }{{\rm Z}}}\,{\phi }_{3}^{{\Upsilon }{{\rm Z}}}\,{\phi }_{4}^{{\Upsilon }{{\rm Z}}}\,{\phi }_{5}^{{\Upsilon }{{\rm Z}}}\,{\phi }_{6}^{{\Upsilon }{{\rm Z}}}\,{\phi }_{7}^{{\Upsilon }{{\rm Z}}}\,{\phi }_{8}^{{\Upsilon }{{\rm Z}}}\,{\phi }_{9}^{{\Upsilon }{{\rm Z}}}\,{\phi }_{10}^{{\Upsilon }{{\rm Z}}}\\ {\phi }_{1}^{{{\rm X}}{\Upsilon }}\,{\phi }_{2}^{{{\rm X}}{\Upsilon }}\,{\phi }_{3}^{{{\rm X}}{\Upsilon }}\,{\phi }_{4}^{{{\rm X}}{\Upsilon }}\,{\phi }_{5}^{{{\rm X}}{\Upsilon }}\,{\phi }_{6}^{{{\rm X}}{\Upsilon }}\,{\phi }_{7}^{{{\rm X}}{\Upsilon }}\,{\phi }_{8}^{{{\rm X}}{\Upsilon }}\,{\phi }_{9}^{{{\rm X}}{\Upsilon }}\,{\phi }_{10}^{{{\rm X}}{\Upsilon }}\end{array}\,)\,$$

Finally, a sensitivity analysis regarding the impact of small variations in the choice of the 14 critical points on the angles was performed. With the use of a Matlab algorithm, all coordinates were randomly translated by up to 1 mm in space and the effect on the angles was measured. The process was repeated 100 times, to simulate the inter-variance of measurements taken by different users.

### Aortic dimensions

**Figure 5:**
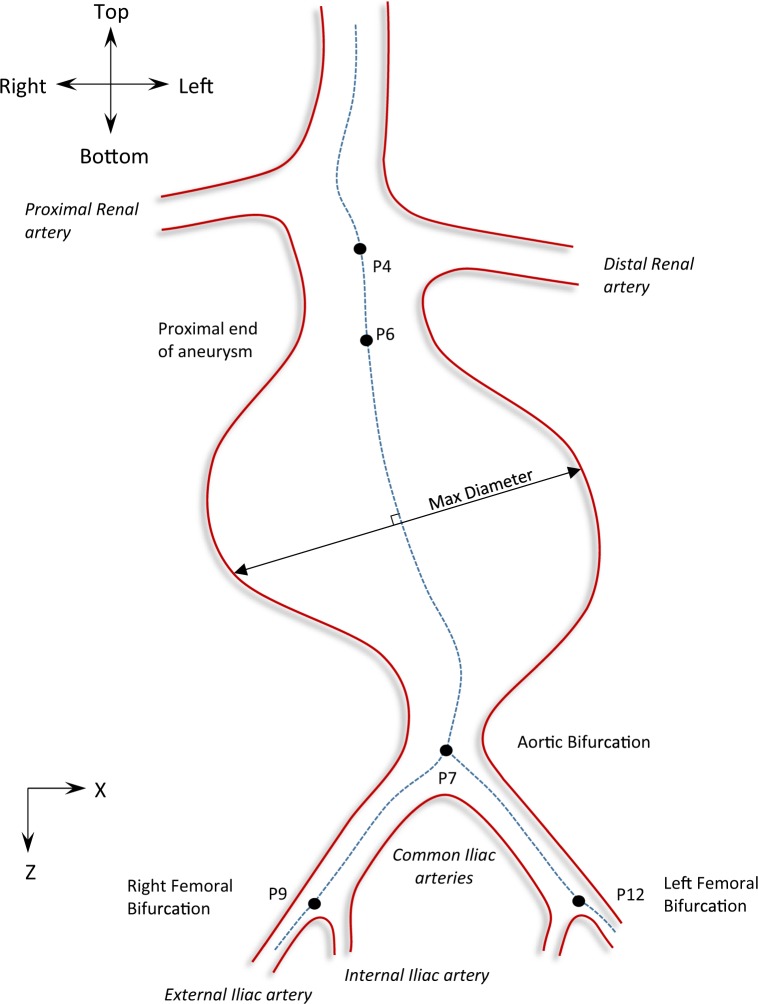
Schematic of the front view of an AAA. XZ-plane. 5 points are identified on the centreline of the aneurysm (dotted line). The maximum diameter is also illustrated.

Regarding AAA dimensions, 9 variables were considered per patient (Table 3 and Fig. 5). Opposite to the previous section, these measurements were conducted by trained technicians of M2S, as part of the initial clinical investigation. Nevertheless, to address any possible limitations of the study, several CTA scans were examined by the authors as well, verifying that the dimensional measurements provided could be accurately reproduced.

**Table 3:** The dimensional variables examined in the AAA geometry. Note that centreline distance refers to the length between two points, measured on the centreline curve (Fig. 5).

Average Neck Diameter	The mean value of two neck diameters, one at the distal renal artery (P4) and one at the most distal extent of the proximal neck (P6).
Neck Length	Centreline distance of the proximal aortic neck (length between points P4 and P6).
Max Diameter	Maximum diameter of the aneurysmal sac.
Distal Diameter	Diameter of the minimum cross section of the aorta below the aneurysm and above the aortic bifurcation (around P7).
Renal to Bi Length	Centreline length from the most distal renal artery (P4) to the aortic bifurcation (P7).
Volume	Volume of the AAA, from the distal renal artery (P4) to the aortic bifurcation (P7).
Average Tortuosity Index	Tortuosity index *T* is defined as $T=\frac{{L}_{1}}{{L}_{2}}\,\,$with *L*_1_ being the centreline distance and *L*_2_ the straight-line distance between 2 points. Herein, the mean value of two tortuosity indexes is calculated, one between the lowest renal artery (P4) and the right femoral bifurcation (P9) and one between the lowest renal artery (P4) and the left femoral bifurcation (P12).
Right Iliac Landing Diameter	The average diameter of the right iliac artery (section of interest: from the aortic bifurcation (P7) to the right femoral bifurcation (P9)).
Left Iliac Landing Diameter	The average diameter of the left iliac artery (section of interest: from the aortic bifurcation (P7) to the left femoral bifurcation (P12)).

All diameters were measured on CTA slices orthogonal to the centreline of the vessel and were calculated to the intima. Diameters have an accuracy of ±1 mm apart from the maximum one (±1.5 mm). The neck length has an accuracy of ±1.5 mm and the renal to Bi length ±2.5 mm. Finally, the volume is expressed in a 5% maximum range error. It should also be noted that 9 tortuosity values were missing due to limitations in the CTA scan images (either the right or left femoral bifurcation was not included in the scans); these values were treated as missing during the statistical analysis.

### Statistical analysis

The collected data were analysed using SPSS (version 25). Normality was tested according to the Shapiro-Wilk test at the 0.05 significance level. Correlation between the examined variables was investigated with a 2-tailed Pearson correlation test at the 0.01 significance level. Lastly, the difference of medians between the genders, as well as symmetry propositions, were examined with the non-parametric, median test for 2 independent medians at the 0.05 significance level.

## Results

### Aortic angles

**Figure 6:**
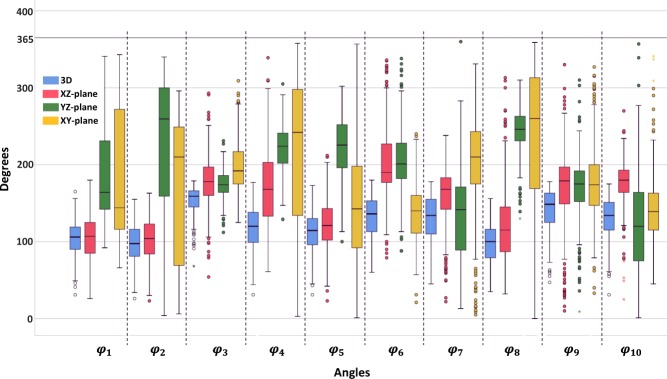
Boxplots of the angles investigated, in their 3D and 2D manifestations.

The median, the interquartile range (IQR) and range of all angles in their 3D and 2D manifestations are reported in Tables 4 and 5. With the exception of a few variables (*φ*_1_, *φ*_2_, *φ*_4_, *φ*_8_, in 3D space and *φ*_4_ in YZ-plane as well as *φ*_6_ in XY-plane), no angles were found to follow the normal distribution. The same was true for the dimensional variables as well (Table 6). It was this observation that lead to the decision to study the medians instead of the means of each variable for a more representative quantification, since medians are resistant to outliers. Figure 6 provides boxplots of all the angles, illustrating the significantly greater range of values for the projected angles compared to the 3D ones.

**Table 4:** The median, the IQR and the range of all measured angles in 3D space (in deg).

Angles in 3D space	Median	IQR	Range
*φ*_1_	106	28	31–165
*φ*_2_	98	36	26–155
*φ*_3_	159	21	68–179
*φ*_4_	120	40	31–177
*φ*_5_	115	34	31–173
*φ*_6_	136	40	60–180
*φ*_7_	134	45	45–178
*φ*_8_	100	38	35–156
*φ*_9_	149	36	47–178
*φ*_10_	134	36	31–175

**Table 5:** The median, the IQR and the range of the projected angles on 3 planes (in deg).

Angles	XZ-plane			YZ-plane			XY-plane		
	Median	IQR	Range	Median	IQR	Range	Median	IQR	Range
*φ*_1_	107	41	26–180	164	87	92–341	147	156	66–350
*φ*_2_	105	40	23–163	259	141	4–340	209	178	6–296
*φ*_3_	178	37	54–293	174	23	112–231	192	43	125–309
*φ*_4_	168	70	61–339	224	39	129–305	242	167	3–358
*φ*_5_	121	40	23–212	225	57	100–302	143	106	1–357
*φ*_6_	190	51	79–336	201	46	88–338	140	49	21–240
*φ*_7_	168	41	22–238	143	83	13–360	210	66	5–331
*φ*_8_	115	58	32–313	246	32	130–310	260	143	0–359
*φ*_9_	179	47	10–330	175	40	9–310	174	53	33–327
*φ*_10_	180	29	25–270	120	88	1–357	139	48	45–341

**Table 6:** The p-values of the normality test of all the variables examined (bold for rejecting the null hypothesis).

Variable	*φ*_1_	*φ*_1_	*φ*_1_	*φ*_1_	*φ*_1_	*φ*_1_	*φ*_1_	*φ*_1_	*φ*_1_	*φ*_1_
**p-value**	0.270	0.272	**0.000**	0.076	**0.000**	**0.000**	**0.000**	0.189	**0.000**	**0.000**
**Variable**	${{\boldsymbol{\phi }}}_{{\bf{1}}}^{{\boldsymbol{{\rm X}}}{\boldsymbol{{\rm Z}}}}$	${{\boldsymbol{\phi }}}_{{\bf{2}}}^{{\boldsymbol{{\rm X}}}{\boldsymbol{{\rm Z}}}}$	${{\boldsymbol{\phi }}}_{{\bf{3}}}^{{\boldsymbol{{\rm X}}}{\boldsymbol{{\rm Z}}}}$	${{\boldsymbol{\phi }}}_{{\bf{4}}}^{{\boldsymbol{{\rm X}}}{\boldsymbol{{\rm Z}}}}$	${{\boldsymbol{\phi }}}_{{\bf{5}}}^{{\boldsymbol{{\rm X}}}{\boldsymbol{{\rm Z}}}}$	${{\boldsymbol{\phi }}}_{{\bf{6}}}^{{\boldsymbol{{\rm X}}}{\boldsymbol{{\rm Z}}}}$	${{\boldsymbol{\phi }}}_{{\bf{7}}}^{{\boldsymbol{{\rm X}}}{\boldsymbol{{\rm Z}}}}$	${{\boldsymbol{\phi }}}_{{\bf{8}}}^{{\boldsymbol{{\rm X}}}{\boldsymbol{{\rm Z}}}}$	${{\boldsymbol{\phi }}}_{{\bf{9}}}^{{\boldsymbol{{\rm X}}}{\boldsymbol{{\rm Z}}}}$	${{\boldsymbol{\phi }}}_{{\bf{10}}}^{{\boldsymbol{{\rm X}}}{\boldsymbol{{\rm Z}}}}$
**p-value**	**0.009**	**0.006**	**0.001**	**0.001**	**0.026**	**0.000**	**0.000**	**0.000**	**0.000**	**0.000**
**Variable**	${{\boldsymbol{\phi }}}_{{\bf{1}}}^{{\boldsymbol{Y}}{\boldsymbol{{\rm Z}}}}$	${{\boldsymbol{\phi }}}_{{\bf{2}}}^{{\boldsymbol{Y}}{\boldsymbol{{\rm Z}}}}$	${{\boldsymbol{\phi }}}_{{\bf{3}}}^{{\boldsymbol{Y}}{\boldsymbol{{\rm Z}}}}$	${{\boldsymbol{\phi }}}_{{\bf{4}}}^{{\boldsymbol{Y}}{\boldsymbol{{\rm Z}}}}$	${{\boldsymbol{\phi }}}_{{\bf{5}}}^{{\boldsymbol{Y}}{\boldsymbol{{\rm Z}}}}$	${{\boldsymbol{\phi }}}_{{\bf{6}}}^{{\boldsymbol{Y}}{\boldsymbol{{\rm Z}}}}$	${{\boldsymbol{\phi }}}_{{\bf{7}}}^{{\boldsymbol{Y}}{\boldsymbol{{\rm Z}}}}$	${{\boldsymbol{\phi }}}_{{\bf{8}}}^{{\boldsymbol{Y}}{\boldsymbol{{\rm Z}}}}$	${{\boldsymbol{\phi }}}_{{\bf{9}}}^{{\boldsymbol{Y}}{\boldsymbol{{\rm Z}}}}$	${{\boldsymbol{\phi }}}_{{\bf{10}}}^{{\boldsymbol{Y}}{\boldsymbol{{\rm Z}}}}$
**p-value**	**0.000**	**0.000**	**0.007**	0.814	**0.000**	**0.000**	**0.000**	**0.000**	**0.000**	**0.000**
**Variable**	${{\boldsymbol{\phi }}}_{{\bf{1}}}^{{\boldsymbol{XY}}}$	${{\boldsymbol{\phi }}}_{{\bf{2}}}^{{\boldsymbol{XY}}}$	${{\boldsymbol{\phi }}}_{{\bf{3}}}^{{\boldsymbol{XY}}}$	${{\boldsymbol{\phi }}}_{{\bf{4}}}^{{\boldsymbol{XY}}}$	${{\boldsymbol{\phi }}}_{{\bf{5}}}^{{\boldsymbol{XY}}}$	${{\boldsymbol{\phi }}}_{{\bf{6}}}^{{\boldsymbol{XY}}}$	${{\boldsymbol{\phi }}}_{{\bf{7}}}^{{\boldsymbol{XY}}}$	${{\boldsymbol{\phi }}}_{{\bf{8}}}^{{\boldsymbol{XY}}}$	${{\boldsymbol{\phi }}}_{{\bf{9}}}^{{\boldsymbol{XY}}}$	${{\boldsymbol{\phi }}}_{{\bf{10}}}^{{\boldsymbol{XY}}}$
**p-value**	**0.000**	**0.000**	**0.000**	**0.000**	**0.000**	0.481	**0.000**	**0.000**	**0.000**	**0.000**
**Variable**	**Average Neck Diameter**	**Neck Length**	**Max Diameter**	**Distal Diameter**	**Renal to Bi Length**	**Volume**	**Average Tortuosity Index**	**Right Iliac Landing Diameter**	**Left Iliac Landing Diameter**	
**p-value**	**0.002**	**0.000**	**0.000**	**0.000**	**0.001**	**0.000**	**0.000**	**0.000**	**0.000**

Lastly, the sensitivity analysis in the choice of the 14 critical points demonstrated small angle alterations, with the median discrepancy being below 0.3% for all angles (Table 7). The average minimum difference of all angles was just 0.01%, while the average maximum was 11.40%. Generally, the discrepancy was higher in the angles *φ*_1_and *φ*_2_ as these angles are calculated via points that are close to each other, hence offset variations of those coordinates result in larger angle alterations.

**Table 7:** The median (and the IQR) of the percentage discrepancy of the resulting 3D angles, when each critical point is translated by up to 1 mm in space, and compared to the original measurement.

*φ*_1_	−0.104 (4.197) %
*φ*_2_	−0.040 (4.099) %
*φ*_3_	−0.221 (2.702) %
*φ*_4_	−0.043 (2.659) %
*φ*_5_	−0.043 (2.556) %
*φ*_6_	−0.110 (2.795) %
*φ*_7_	−0.162 (3.287) %
*φ*_8_	−0.017 (2.665) %
*φ*_9_	−0.158 (2.750) %
*φ*_10_	−0.166 (3.444) %

### Aortic dimensions

The median, the IQR and range of all dimensional variables examined is reported in Table 8. Note that the iliacs are required to have a minimum diameter of usually 7 mm for the delivery of the endograft^2^, a requirement that herein was fulfilled since all of the examined patients underwent EVAR.

**Table 8:** The median, the IQR and range of the dimensional variables.

Variable	Median	IQR	Range
Average Neck Diameter (mm)	23.1	3.9	16.8–34.2
Neck Length (mm)	22.0	20.0	2.0–50.0
Max Diameter (mm)	52.0	8.5	40.3–86.5
Distal Diameter (mm)	18.0	6.7	10.5–46.5
Renal to Bi Length (mm)	123.9	20.3	93.4–181.1
Volume ± 5% (cm^−3^)	127.0	55.0	54.1–454.6
Average Tortuosity Index	1.22	0.11	1.07–1.65
Right Iliac Landing Diameter (mm)	13.5	3.3	7.6–28.7
Left Iliac Landing Diameter (mm)	13.0	3.4	6.7–36.1

### Statistical analysis

The first proposition to be tested was the lateral and anterior-posterior symmetry of the AAA. In general, AAAs appear to be significantly asymmetric and herein, the medians of the pairs of angles *φ*_1_– *φ*_2_, *φ*_5_– *φ*_8_, *φ*_6_– *φ*_9_ and *φ*_7_ –*φ*_10_ were compared. The analysis showed that only *φ*_7_–*φ*_10_ in 3D space, and *φ*_1_– *φ*_2_ and *φ*_5_– *φ*_8_ on the XZ-plane have statistically insignificant differences. For all other angles and angle projections, the left side of the AAA was different to the right one. Particularly for *φ*_3_ and *φ*_4_ on the XZ-plane, medians were <180°, creating a slight, general trend of neck angulation to the right. Regarding the YZ-plane, *φ*_4_ had a median of 224°, showing a greater anterior expansion, similar to *φ*_5_ (225°).

A few angles appeared to have weak linear correlations with coefficients ranging between 0.1 and 0.3. The only pair with a moderate correlation though, was *φ*_1_–*φ*_2_, with a correlation value of 0.542, suggesting that when the angle of one renal increases, the angle of the other will have a tendency to increase as well.

Subsequently, a series of correlations were tested for the dimensional variables of the study. Moderate correlations emerged between the pairs Volume – Average Tortuosity Index (coefficient ρ = 0.299), Age – Average Tortuosity Index (coefficient ρ = 0.399), Volume – Aneurysmal Length (coefficient ρ = 0.590) and Right Iliac Landing Diameter – Left Iliac Landing Diameter (coefficient ρ = 0.612). Finally, the variables Volume – Maximum Diameter were strongly correlated (coefficient ρ = 0.814).

The effect of gender was also examined. In the literature, body size information (i.e. height and weight) have been reported to influence and explain reasons for gender differences and are often employed in such comparisons^16,17^. Nevertheless, because such data were not available herein, all dimensional variables were indexed to (i.e. divided by) Average Neck Diameter to acquire normalized data. Via this process, it was identified that the normalized maximum diameter of the aneurysm was statistically significantly bigger in males as well as the normalized Renal to Bi length and the normalized Average Tortuosity Index (Table 9). It is interesting to note that in a sensitivity analysis, during which the measurement data were altered inside their error margin, the normalized maximum diameter was not always statistically significantly different for the two genders, yet the difference in the other two variables remained. Regarding the angles in 3D space, *φ*_2_ appeared statistically significantly different between the genders (99° vs 88° for males and females respectively), similarly to *φ*_4_ (119° vs 109°) and *φ*_6_ (132° vs 145°) (Table 10).

**Table 9:** p-values of the nonparametric test examining differences in the medians of the normalized dimensional variables across the genders (bold for rejecting the null hypothesis).

Variable	Neck Length	Max Diameter	Distal Diameter	Renal to Bi Length	Volume	Average Tortuosity Index	Right Iliac Landing Diameter	Left Iliac Landing Diameter
**p-value**	0.097	**0.043**	0.854	**0.006**	0.357	**0.014**	0.233	0.197

**Table 10:** p-values of the nonparametric test examining differences in the medians of the 3D angles across the genders (bold for rejecting the null hypothesis).

Variable	*φ*_1_	*φ*_2_	*φ*_3_	*φ*_4_	*φ*_5_	*φ*_6_	*φ*_7_	*φ*_8_	*φ*_9_	*φ*_10_
**p-value**	0.990	**0.026**	0.614	**0.045**	0.873	**0.001**	0.308	0.873	0.912	0.067

Histograms of all the (non-normalized) variables are provided in the Supplementary Information.

## Discussion

Despite the fact that the geometrical complexity of the aneurysmal site is highly influenced by the angles of the aorta, the renal arteries and the iliacs, documentation of them is extremely rare in the literature. In this retrospective study, the aim was to identify all major angles involved in an AAA, to allow the description and reproduction of its shape. At the same time, common dimensional measurements were recorder and statistically analysed. The data used were obtained from a clinical study of the Anaconda^TM^ endograft. This device is considered to be able to tackle severe angulations and is used for challenging EVAR cases^18^, hence choosing to acquire data from the specific clinical trial was regarded appropriate, in order to examine a wide range of geometries.

The angle set being proposed herein is a major extension to the angles commonly used. Both in the literature and specialized clinical software, attention is given to the neck angles alone, herein called *φ*_3_ and *φ*_4_. By mapping the entire AAA region though, a unique insight into the pathology has been achieved. The extreme values identified (see for example the very low values of *φ*_4_ in 3D space or of *φ*_4_ and *φ*_8_ in the XZ-plane) could be used by stent graft manufacturers to build (experimental or numerical) AAA case studies that would allow them to identify the operating range of their endografts. Moreover, the lack of strong correlation between aortic angles suggest that an investigator is free to construct any extreme AAA case-study with the values provided in Tables 4–5 in a mix-and-match manner. This strategy can have a powerful effect in EVAR stent graft designing. Looking further into the future, surgeons could eventually perform measurements on challenging patient CTA scans and decide which EVAR device could tackle best a specific geometry according to the Instructions for Use of each manufacturer.

It is important to acknowledge that from a clinical perspective, not all variables are equally significant for EVAR. Angles *φ*_1_ and *φ*_2_ relate to the renals and become relevant only when the aneurysmal neck is too short and a fenestrated endograft needs to be used. On the contrary, angles *φ*_3_ and *φ*_4_ are always important since greater neck angulation increases endoleak flow^19^. Li *et al*.^20^ suggested that when the aortic neck (herein *φ*_3_) is lower than 145°, devices with high fixation forces should be used. They also mention that the anterior-posterior neck angle (*φ*_3_ on the YZ-plane) influences the migration force of the stent graft significantly. The lower angles studied (i.e angles *φ*_5_ – *φ*_10_) refer to the iliac arteries and are critical for the successful delivery of the endograft. If too acute, open aortic surgery should be preferred.

It should be highlighted that the CTA scans obtained for this study reflect a specific group of AAA patients, i.e. those who undergo EVAR. As a result, the conclusions drawn should be generalized to the overall AAA population with caution. Ideally, an equivalent set of angle values corresponding to the healthy population should also be pursued. Such a set would serve as reference for the reported results. Moreover, it would allow a more holistic view of the pathology and its effect on the aorta. In future, a comparison between the healthy and the aneurysmal aortic shape should be conducted and possibly shed light into the development of the disease.

In this study, no dimensional and almost no angular variables followed a normal distribution. Frank *et al*.^15^ reported a similar finding when examining the maximum AAA diameter. In agreement to the results presented here, Wolf *et al*.^5^ have also reported a positive correlation of age and tortuosity and an absence of connection between age and size of the aneurysmal sac. In their study, though, the size of the aneurysm was also unrelated to tortuosity, a result that was not confirmed herein. A similar disagreement is raised with results of Bayle *et al*.^21^ who reported that the bigger the diameter of the aneurysm, the shorter the aneurysmal neck length becomes. No such correlation was observed herein, yet the results of Bayle *et al*. were acquired from the general AAA population. It is also worth noting that both these studies had significantly less patients enrolled (75 and 86 respectively). Finally, the asymmetry of the AAA has been mentioned by other investigators as well^20^.

In the attempt to identify differences in the shape of the AAA between the two genders, 3 angles were found to have statistically significant different medians. It is possible, however, that the number of these angles might be an overestimation because of the multiple post hoc comparative tests, and that similarities are even greater between the two genders.

AAA incidents are four to six times more frequent in men than in women^22^. In the present study, the number of male patients was 6.5 times greater than the number of females, implying that males might have been slightly overrepresented. Nevertheless, the framework built for the angular characterization of the aneurysmal aorta, along with the demonstration that the AAA angles do not, in general, correlate with each other, can bring new insight to the topic. The process developed has allowed the establishment of representative and worst case geometries of AAAs that can be an aid to successful EVAR. The data produced herein can be used to create CAD aortic models for virtual deployment, testing and optimization of endografts during their development phase, or assist the characterization of patient specific AAAs (e.g. as extreme, treatable, or treatable with a specific stent graft). To the authors’ knowledge, this is the most extensive geometrical study of the AAA shape.

## Supplementary information

Statistical distribution of AAA variables

## Data Availability

All datasets associated with this manuscript are available in FIGSHARE (10.6084/m9.figshare.9897632) and from the corresponding author upon reasonable request.

## References

[1] Sethi RKV (2013). Impact of hospital market competition on endovascular aneurysm repair adoption and outcomes. J. Vasc. Surg..

[2] Walker TG (2010). Clinical Practice Guidelines for Endovascular Abdominal Aortic Aneurysm Repair: Written by the Standards of Practice Committee for the Society of Interventional Radiology and Endorsed by the Cardiovascular and Interventional Radiological Society of Europe. J Vasc Interv Radiol.

[3] Ahn SS (1997). Reporting standards for infrarenal endovascular abdominal aortic aneurysm repair. J. Vasc. Surg..

[4] Chaikof EL (2002). Identifying and grading factors that modify the outcome of endovascular aortic aneurysm repair. J. Vasc. Surg..

[5] Wolf Yehuda G., Tillich Manfred, Lee W.Anthony, Rubin Geoffrey D., Fogarty Thomas J., Zarins Christopher K. (2001). Impact of aortoiliac tortuosity on endovascular repair of abdominal aortic aneurysms: Evaluation of 3D computer-based assessment. Journal of Vascular Surgery.

[6] Carpenter JP (2001). Impact of exclusion criteria on patient selection for endovascular abdominal aortic aneurysm repair. J. Vasc. Surg..

[7] Wolf YG (2000). Endovascular repair of abdominal aortic aneurysms: Eligibility rate and impact on the rate of open repair. J. Vasc. Surg..

[8] Armon MP (1997). Anatomical suitability of abdominal aortic aneurysms for endovascular repair. Br. J. Surg..

[9] Kristmundsson, T. A Novel Method to Estimate Iliac Tortuosity in Evaluating EVAR Access. 157–164 (2012). 10.1583/11-3704.1 22545879

[10] Henretta JP (1999). Special iliac artery considerations during aneurysm endografting. Am. J. Surg..

[11] Clough R, Hertault A, Azzaoui R, Sobocinski J, Haulon S (2016). Low-Profile EVAR. Endovasc. today.

[12] Li Z, Kleinstreuer C (2006). Analysis of biomechanical factors affecting stent-graft migration in an abdominal aortic aneurysm model. J. Biomech..

[13] Morris L, Delassus P, Walsh M, McGloughlin T (2004). A mathematical model to predict the *in vivo* pulsatile drag forces acting on bifurcated stent grafts used in endovascular treatment of abdominal aortic aneurysms (AAA). J. Biomech..

[14] U.S. National Library of Medicine. Available at: https://clinicaltrials.gov/ct2/home. 10.1080/15360280801989377 28792816

[15] Frank A. L., *et al*. Variability in measurement of abdominal aortic aneurysms. *J. Vasc. Surg*. 945–952 (1995). 10.1016/s0741-5214(95)70222-9 7776474

[16] Jones GT (2019). Correcting for Body Surface Area Identifies the True Prevalence of Abdominal Aortic Aneurysm in Screened Women. Eur. J. Vasc. Endovasc. Surg..

[17] Matyal R (2015). Impact of gender and body surface area on outcome after abdominal aortic aneurysm repair. Am. J. Surg..

[18] Rödel SGJ, Zeebregts CJ, Huisman AB, Geelkerken RH (2014). Results of the Anaconda endovascular graft in abdominal aortic aneurysm with a severe angulated infrarenal neck. J. Vasc. Surg..

[19] Albertini J-N, Macierewicz JA, Yusuf SW, Wenham PW, Hopkinson BR (2001). Pathophysiology of Proximal Perigraft Endoleak Following Endovascular Repair of Abdominal Aortic Aneurysms: a Study Using a Flow Model. Eur. J. Vasc. Endovasc. Surg..

[20] Li Z, Kleinstreuer C, Farber M (2005). Computational analysis of biomechanical contributors to possible endovascular graft failure. Biomech. Model. Mechanobiol..

[21] Bayle O (1997). Morphologic assessment of abdominal aortic aneurysms by spiral computed tomographic scanning. J. Vasc. Surg..

[22] Starr JE, Halpern V (2013). Abdominal aortic aneurysms in women. J. Vasc. Surg..

