# Supplementary material for: A Methodology to Quantify the Geometrical Complexity of the Abdominal Aortic Aneurysm
Source: Sci Rep. 2019 Nov 22;9:17379. doi: 10.1038/s41598-019-53820-z (PMC6874586; doi:10.1038/s41598-019-53820-z)
Supplement: Supplementary file 1 — Statistical distribution of AAA variables [file 41598_2019_53820_MOESM1_ESM.pdf]

A METHODOLOGY TO QUANTIFY THE GEOMETRICAL COMPLEXITY OF THE ABDOMINAL AORTIC ANEURYSM

Faidon Kyriakou, William Dempster, David Nash

Supplementary Information

Statistical distribution of AAA variables

The statistical distribution of all the variables examined are documented below. Results concern the analysis of 258 AAA patients enrolled in the 2009-2011 study: "Vascutek Anaconda stent graft system phase II IDE study". For variable definitions refer to Tables 1 – 3.

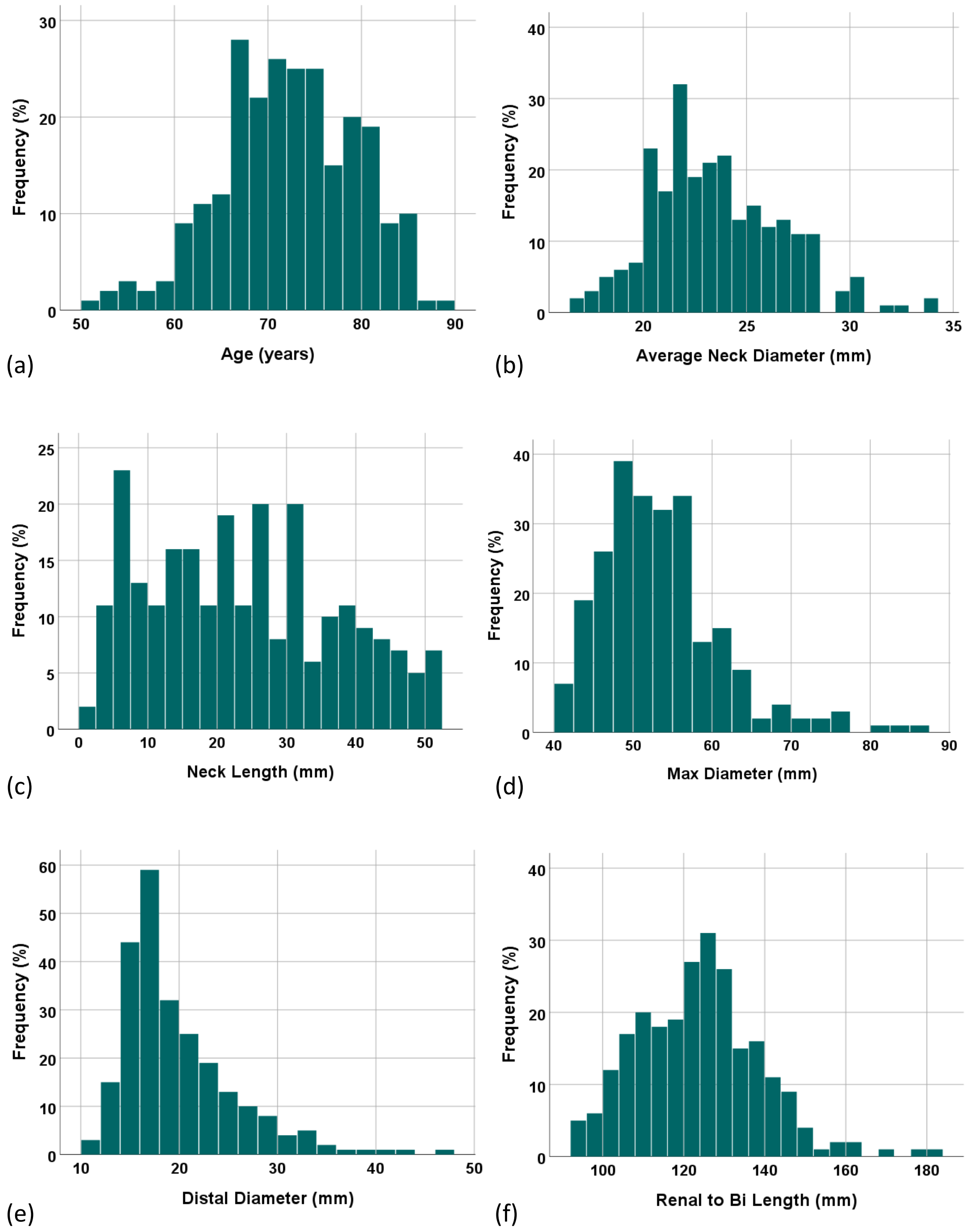

Fig. A.1 Frequency distribution of age (a), average neck diameter (b), neck length (c), maximum diameter (d), distal diameter (e) and renal to bifurcation length (f).

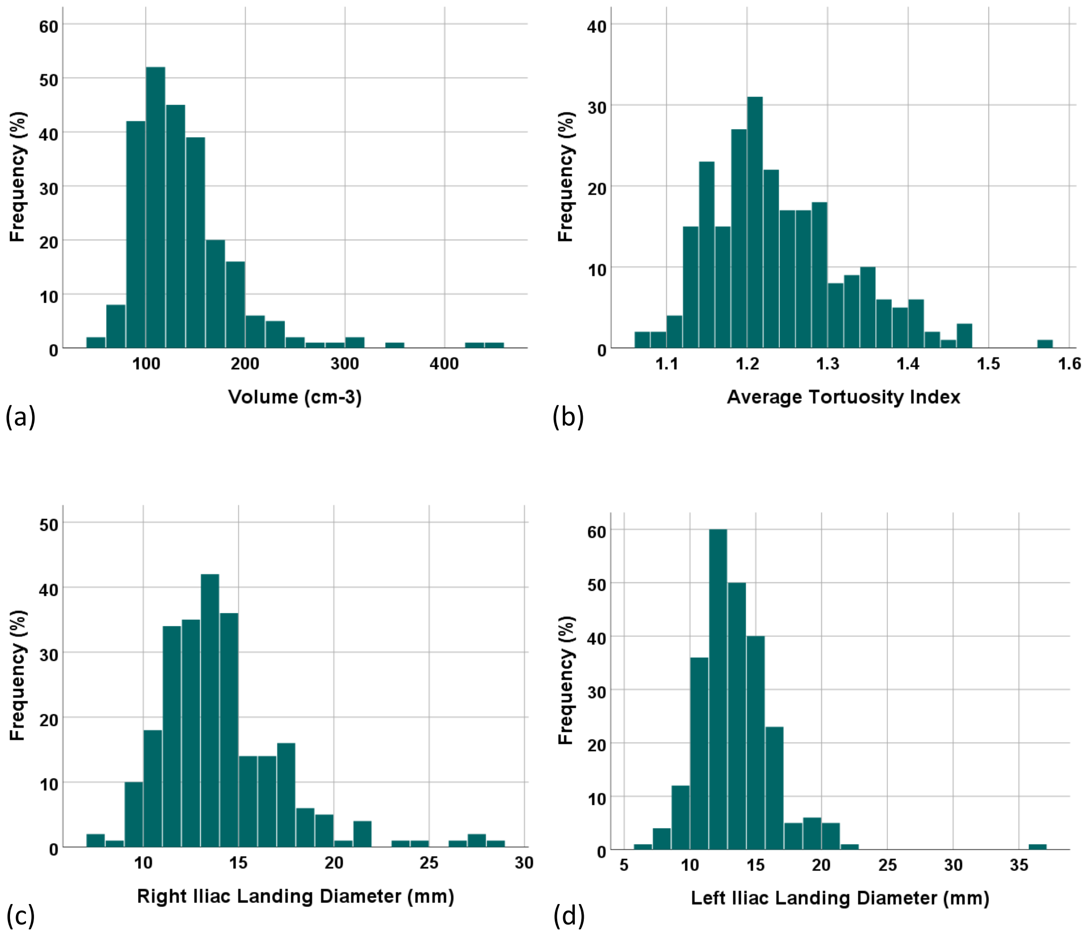

Fig. A.2 Frequency distribution of volume (a), average tortuosity index (b), right iliac landing diameter (c) and left iliac landing diameter (d).

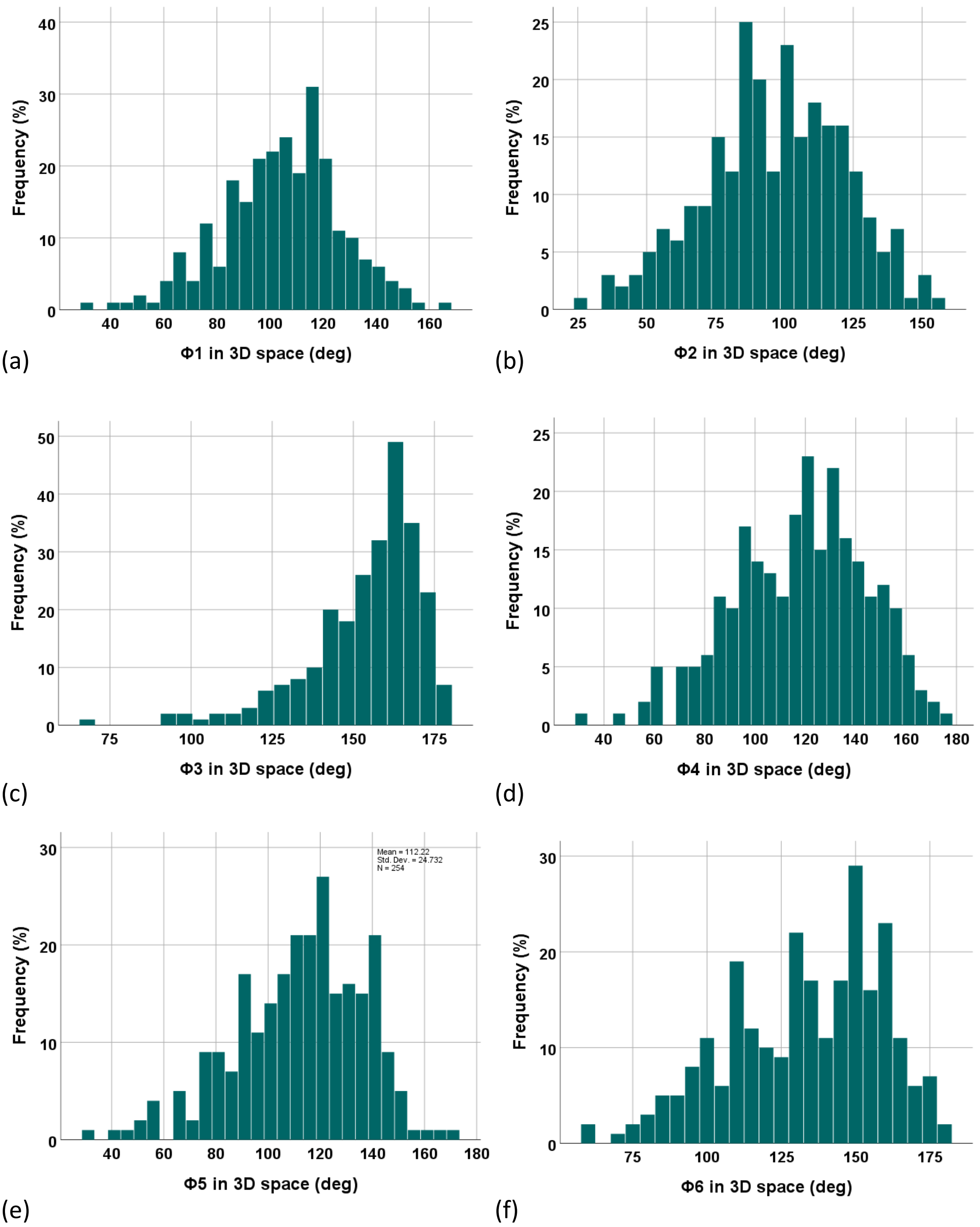

Fig. A.3 Frequency distribution of the angles $\varphi_1 - \varphi_6$ ((a) – (f) respectively) in 3D space.

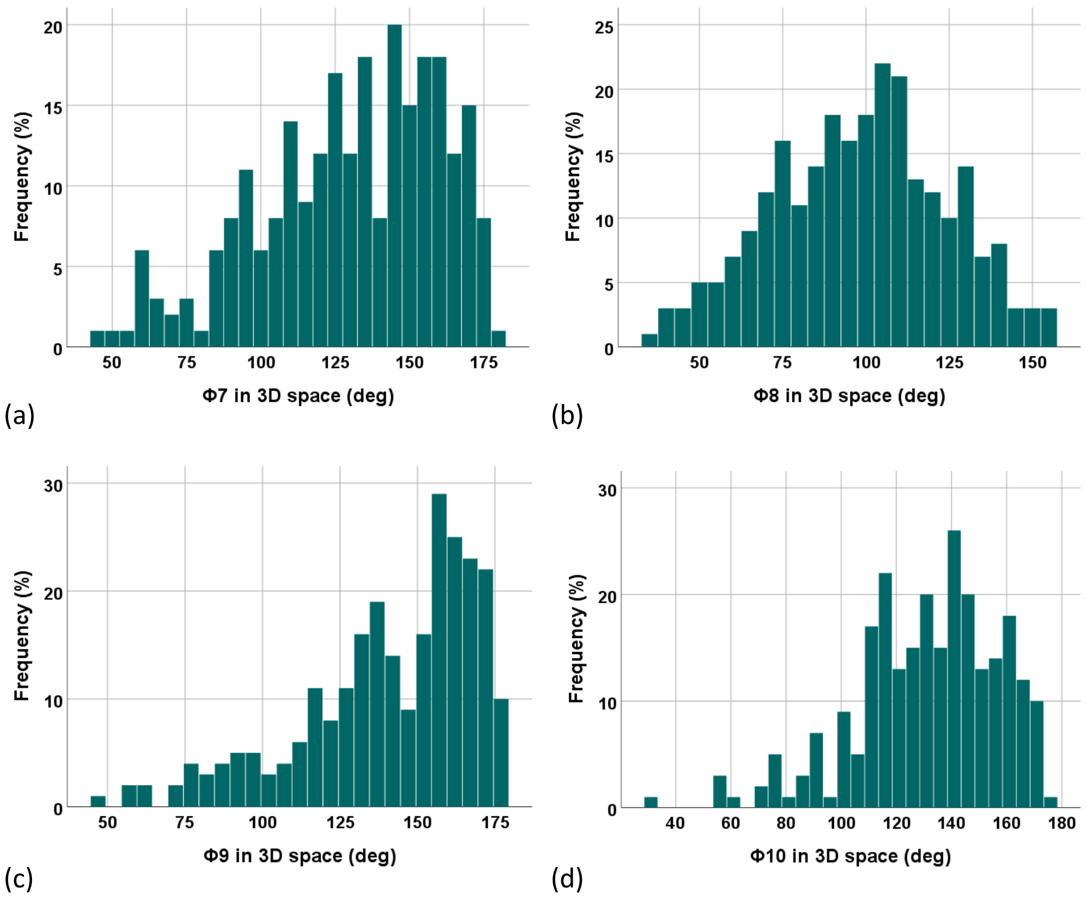

Fig. A.4 Frequency distribution of the angles $\varphi_7 - \varphi_{10}$ ((a) – (d) respectively) in 3D space.

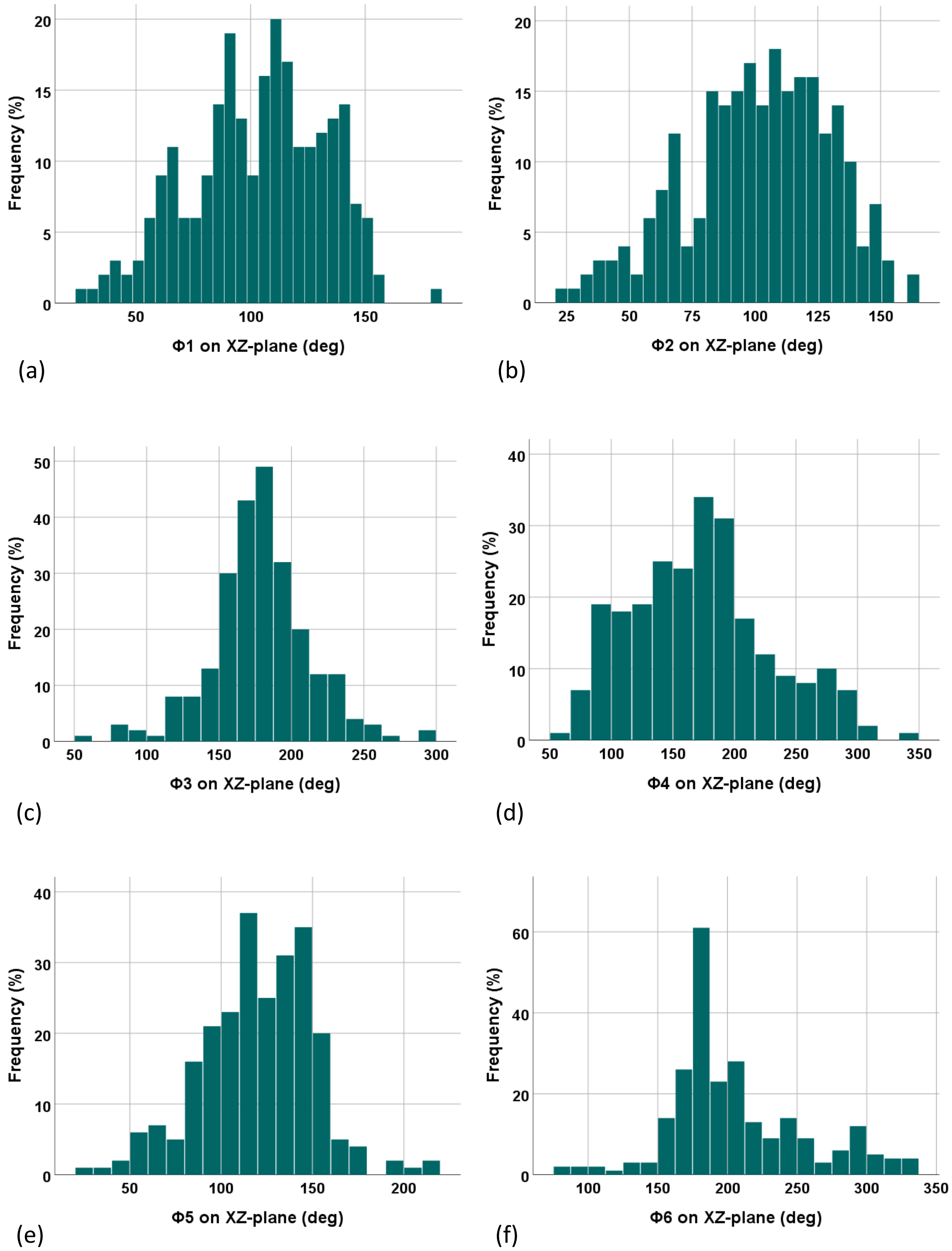

Fig. A.5 Frequency distribution of the angles $\varphi_1 - \varphi_6$ ((a) – (f) respectively) on the XZ-plane.

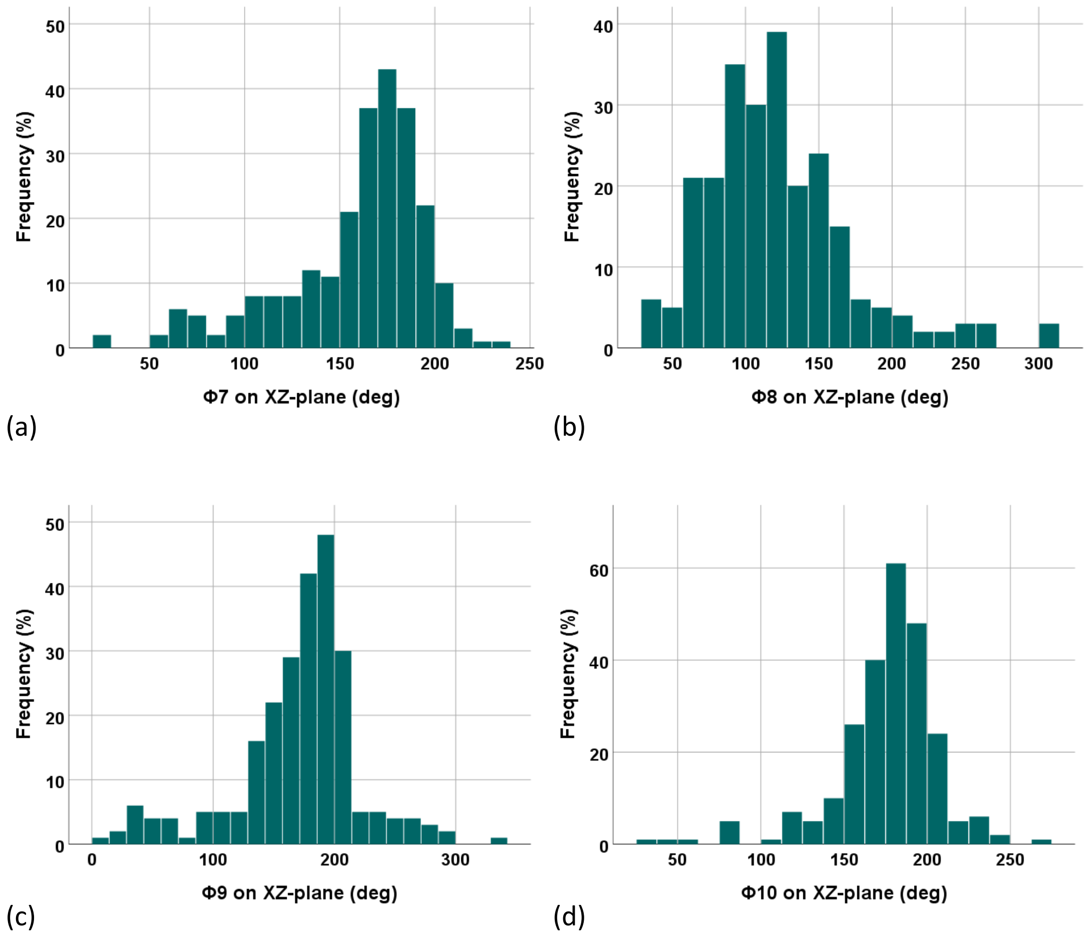

Fig. A.6 Frequency distribution of the angles $\varphi_7 - \varphi_{10}$ ((a) – (d) respectively) on the XZ-plane.

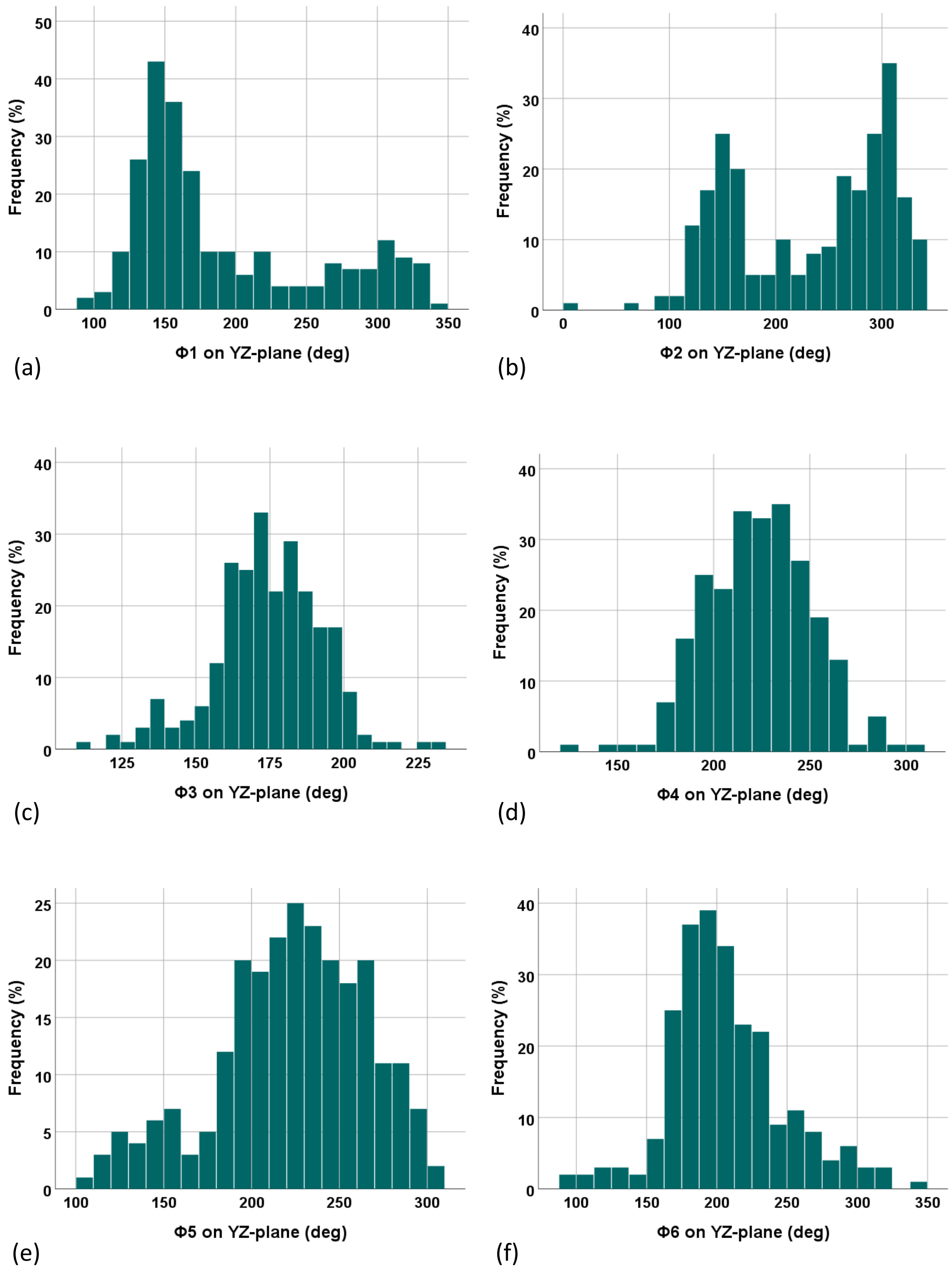

Fig. A.7 Frequency distribution of the angles $\varphi_1 - \varphi_6$ ((a) – (f) respectively) on the YZ-plane.

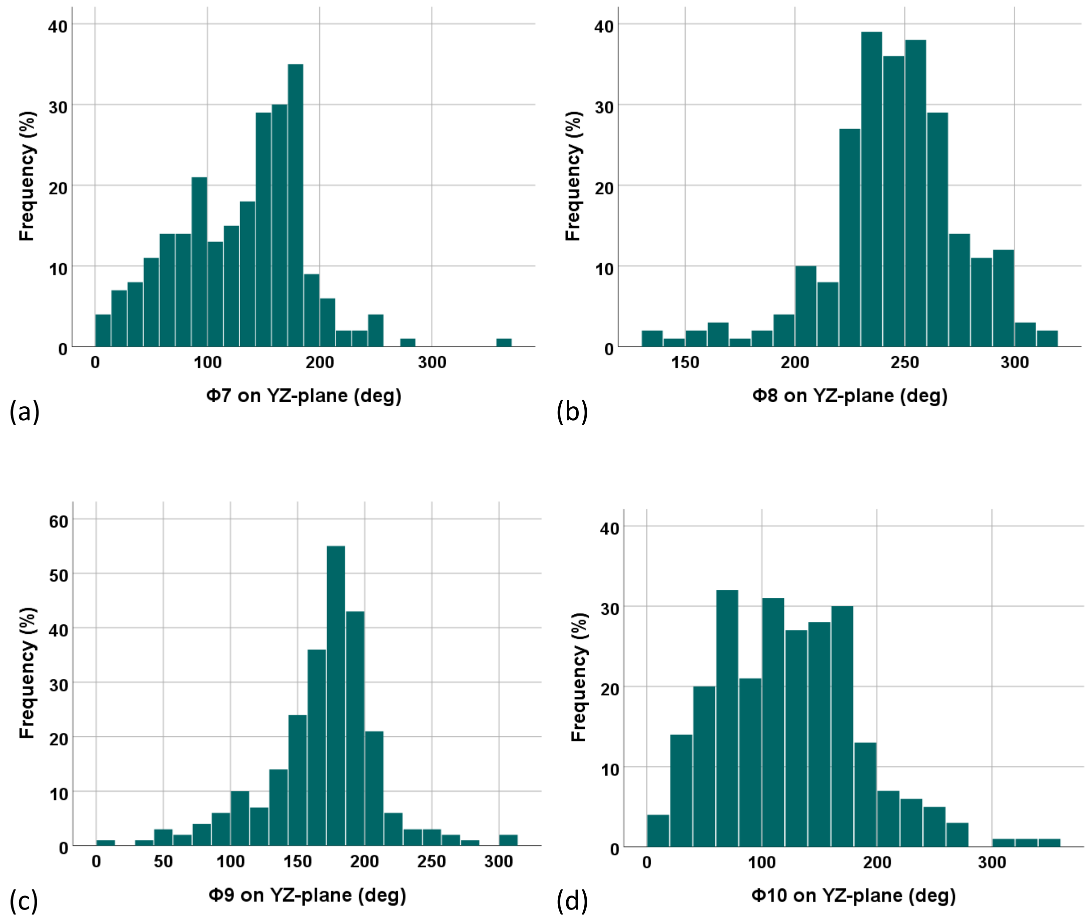

Fig. A.8 Frequency distribution of the angles $\varphi_7 - \varphi_{10}$ ((a) – (d) respectively) on the YZ-plane.

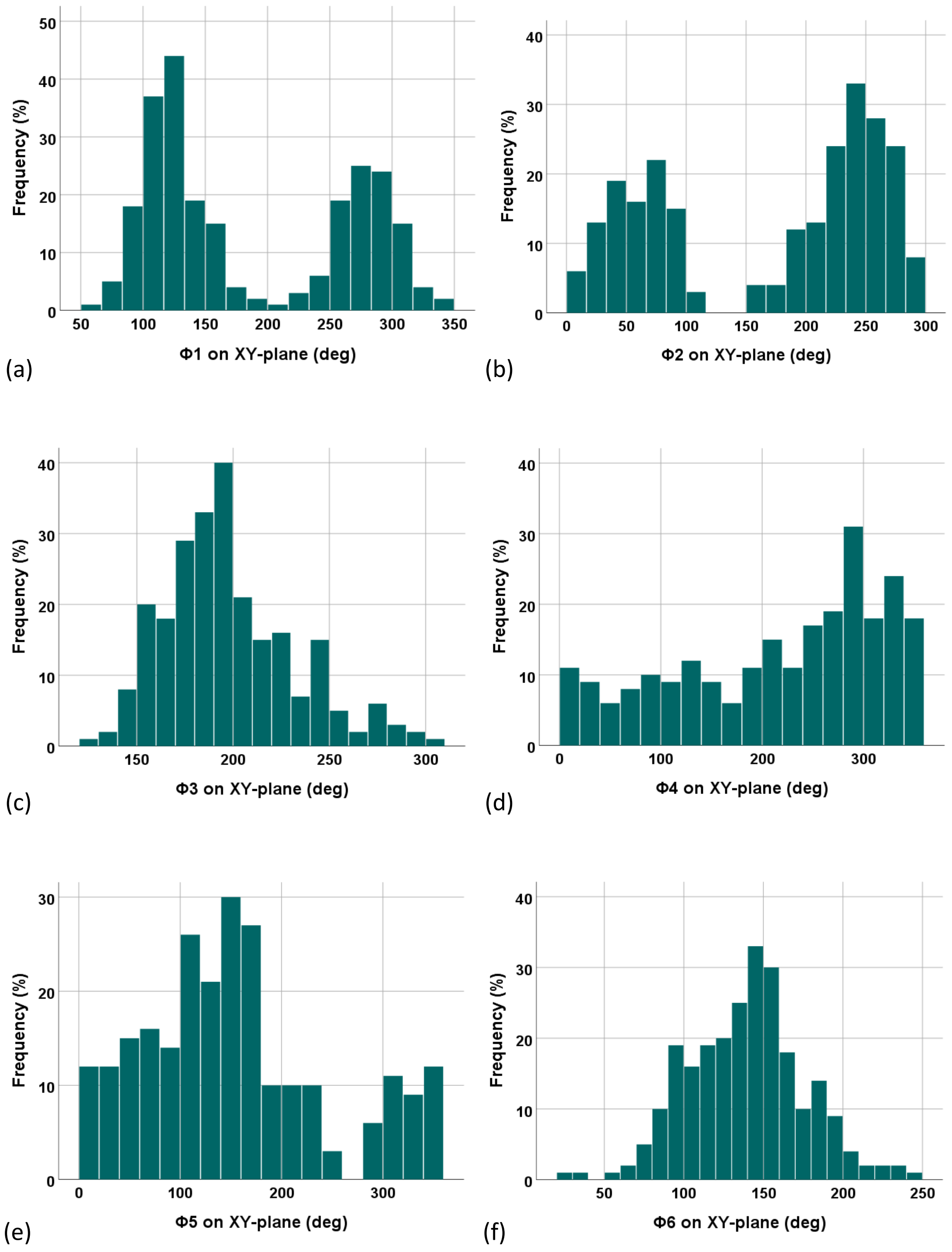

Fig. A.9 Frequency distribution of the angles $\varphi_1 - \varphi_6$ ((a) – (f) respectively) on the XY-plane.

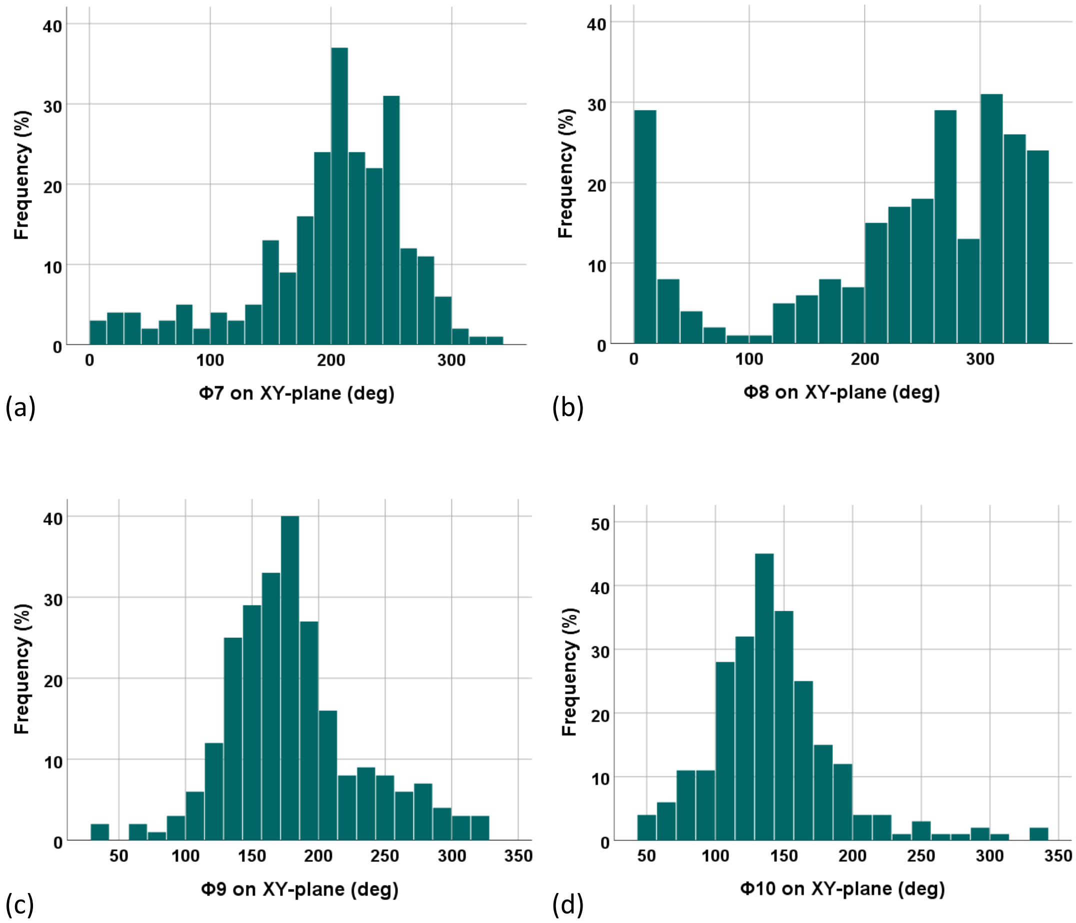

Fig. A.10 Frequency distribution of the angles $\varphi_7 - \varphi_{10}$ ((a) – (d) respectively) on the XY-plane.
